# Author Correction: A ZFYVE21-Rubicon-RNF34 signaling complex promotes endosome-associated inflammasome activity in endothelial cells

**DOI:** 10.1038/s41467-023-39154-5

**Published:** 2023-06-07

**Authors:** Xue Li, Quan Jiang, Guiyu Song, Mahsa Nouri Barkestani, Qianxun Wang, Shaoxun Wang, Matthew Fan, Caodi Fang, Bo Jiang, Justin Johnson, Arnar Geirsson, George Tellides, Jordan S. Pober, Dan Jane-wit

**Affiliations:** 1 VA Connecticut Healthcare System, West Haven, CT USA; 2 Department of Cardiovascular Medicine, Yale University School of Medicine, New Haven, CT USA; 3 Department of Nephrology, Shengjing Hospital of China Medical University, Shenyang, China; 4 Department of Obstetrics and Gynecology, Shengjing Hospital of China Medical University, Shenyang, China; 5 Yale College, Yale University, New Haven, CT USA; 6 Department of Surgery, Yale University School of Medicine, New Haven, CT USA; 7 Department of Vascular Surgery, The First Hospital of China Medical University, Shenyang, China; 8 Department of Immunobiology, Yale University School of Medicine, New Haven, CT USA

**Keywords:** Inflammasome, Transplant immunology, Complement cascade

Correction to: *Nature Communications* 10.1038/s41467-023-38684-2, published online 24 May 2023

In this article the affiliation details for Xue Li were incorrectly given as ‘Dept of Nephrology, The First Hospital of China Medical University, Shenyang, China’, but should have been ‘Department of Nephrology, Shengjing Hospital of China Medical University, Shenyang, China’.

The original article has been corrected.

