# Author Correction: A ZFYVE21-Rubicon-RNF34 signaling complex promotes endosome-associated inflammasome activity in endothelial cells

**DOI:** 10.1038/s41467-023-44225-8

**Published:** 2023-12-18

**Authors:** Xue Li, Quan Jiang, Guiyu Song, Mahsa Nouri Barkestani, Qianxun Wang, Shaoxun Wang, Matthew Fan, Caodi Fang, Bo Jiang, Justin Johnson, Arnar Geirsson, George Tellides, Jordan S. Pober, Dan Jane-wit

**Affiliations:** 1 VA Connecticut Healthcare System, West Haven, CT USA; 2 Department of Cardiovascular Medicine, Yale University School of Medicine, New Haven, CT USA; 3 Department of Nephrology, Shengjing Hospital of China Medical University, Shenyang, China; 4 Department of Obstetrics and Gynecology, Shengjing Hospital of China Medical University, Shenyang, China; 5 Yale College, Yale University, New Haven, CT USA; 6 Dept of Surgery, Yale University School of Medicine, New Haven, CT USA; 7 Dept of Vascular Surgery, The First Hospital of China Medical University, Shenyang, China; 8 Dept of Immunobiology, Yale University School of Medicine, New Haven, CT USA

**Keywords:** Inflammasome, Transplant immunology, Complement cascade

Correction to: *Nature Communications* 10.1038/s41467-023-38684-2, published online 24 May 2023

The original version of this Article did not acknowledge Guiyu Song as a corresponding author. This has now been corrected in both the PDF and HTML versions of the Article.

