# Author Correction: A ZFYVE21-Rubicon-RNF34 signaling complex promotes endosome-associated inflammasome activity in endothelial cells

**DOI:** 10.1038/s41467-026-73044-w

**Published:** 2026-05-28

**Authors:** Xue Li, Quan Jiang, Guiyu Song, Mahsa Nouri Barkestani, Qianxun Wang, Shaoxun Wang, Matthew Fan, Caodi Fang, Bo Jiang, Justin Johnson, Arnar Geirsson, George Tellides, Jordan S. Pober, Dan Jane-wit

**Affiliations:** 1 VA Connecticut Healthcare System, West Haven, CT USA; 2 Department of Cardiovascular Medicine, Yale University School of Medicine, New Haven, CT USA; 3 Department of Nephrology, Shengjing Hospital of China Medical University, Shenyang, China; 4 Department of Obstetrics and Gynecology, Shengjing Hospital of China Medical University, Shenyang, China; 5 Yale College, Yale University, New Haven, CT USA; 6 Dept of Surgery, Yale University School of Medicine, New Haven, CT USA; 7 Dept of Vascular Surgery, The First Hospital of China Medical University, Shenyang, China; 8 Dept of Immunobiology, Yale University School of Medicine, New Haven, CT USA

**Keywords:** Inflammasome, Transplant immunology, Complement cascade

Correction to: *Nature Communications* 10.1038/s41467-023-38684-2, published online 24 May 2023

In the version of this article initially published, there was a presentation error in Fig. 1c, where the upper-left quadrant image was a duplicate of the lower-right quadrant. In the Methods, a section on protocols for staining tissue biopsies, “Cyclic immunofluorescence,” was not included. The figure and Methods are now amended in the HTML and PDF versions of the article.

