# Supplementary material for: A ZFYVE21-Rubicon-RNF34 signaling complex promotes endosome-associated inflammasome activity in endothelial cells
Source: Nat Commun. 2023 May 24;14:3002. doi: 10.1038/s41467-023-38684-2 (PMC10209169; doi:10.1038/s41467-023-38684-2)
Supplement: Supplementary file 1 — Supplementary Information [file 41467_2023_38684_MOESM1_ESM.pdf]

SUPPLEMENTARY INFORMATION

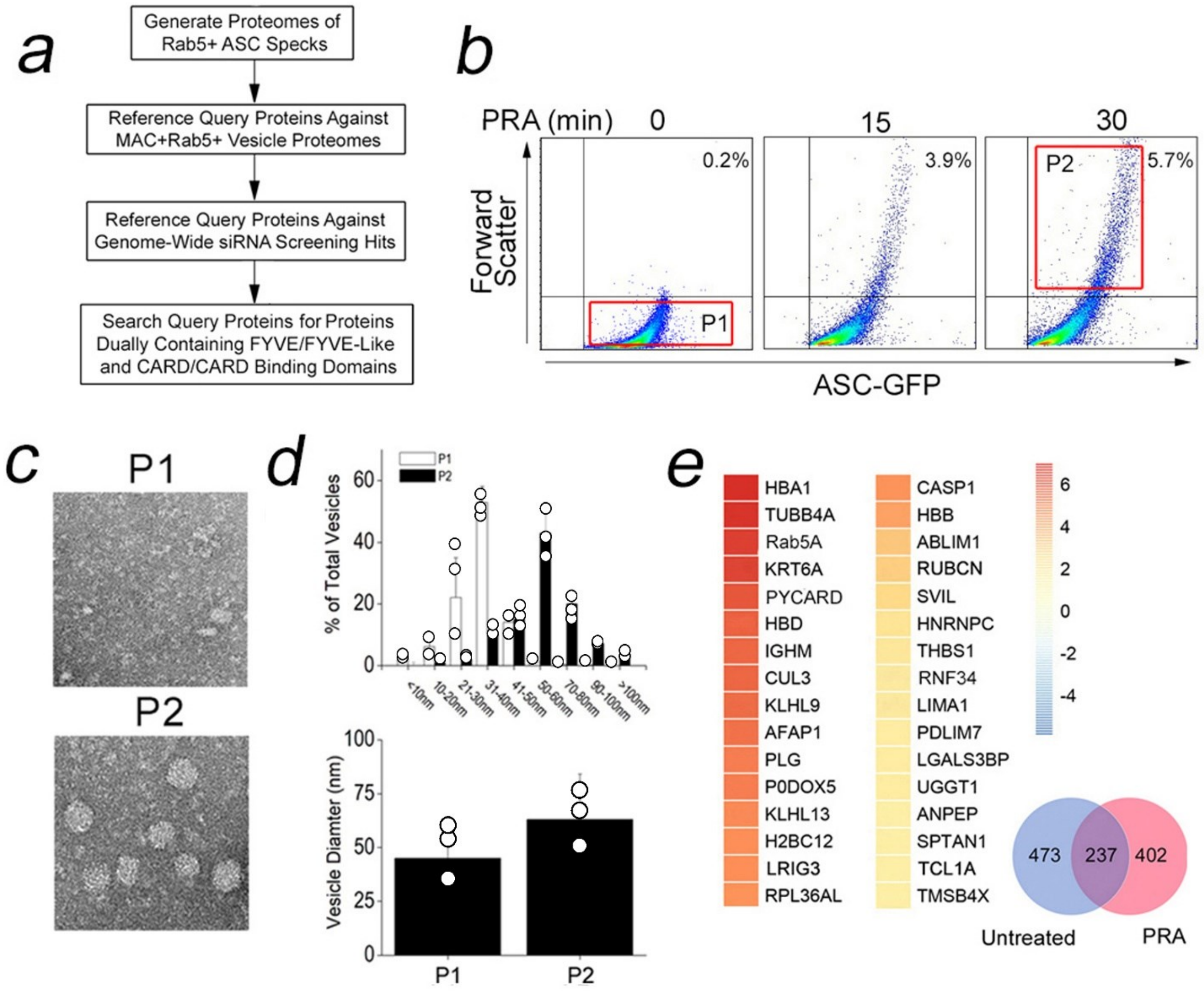

Supplementary Figure 1. A Non-Biased Approach for Identifying Proteins Involved in Endosome-Associated Inflammasome Activity. Iterative search algorithm to identify new Rab5-associated proteins modulating inflammasome activity (*a*). HUVECs were stably transduced with ASC-GFP, treated with PRA for 30 min, sonicated, and subcellular contents were ultracentrifuged, gated, and Forward Scatter^{hi}GFP+ events were isolated by FACS sorting (*b*). FACS-sorted vesicles were analyzed by electron microscopy (*c,d*) and subjected to proteomic analysis by LC-MS/MS (*e*). Proteomic analyses were performed using 3 separate HUVEC donors. Data are presented as mean values +/- SD.

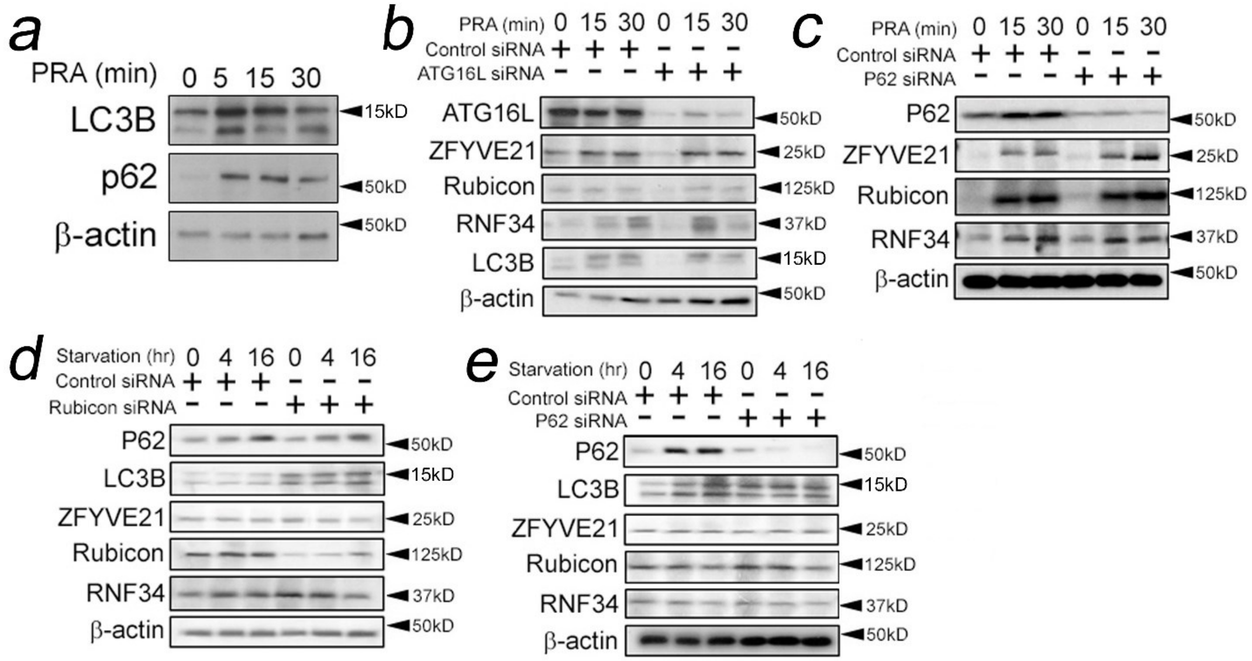

Supplementary Figure 2. HUVECs were analyzed by Western blot analysis following treatment with PRA (*a*). HUVECs were transfected control siRNA and siRNA against ATG16L (*b*) or P62 (*c*) prior to treatment with PRA. HUVECs were serum starved for the times indicated (*d*). HUVECs were transfected with P62 siRNA prior to serum starvation for the times indicated (*e*). Experiments were repeated 3 times using separate HUVEC donors.

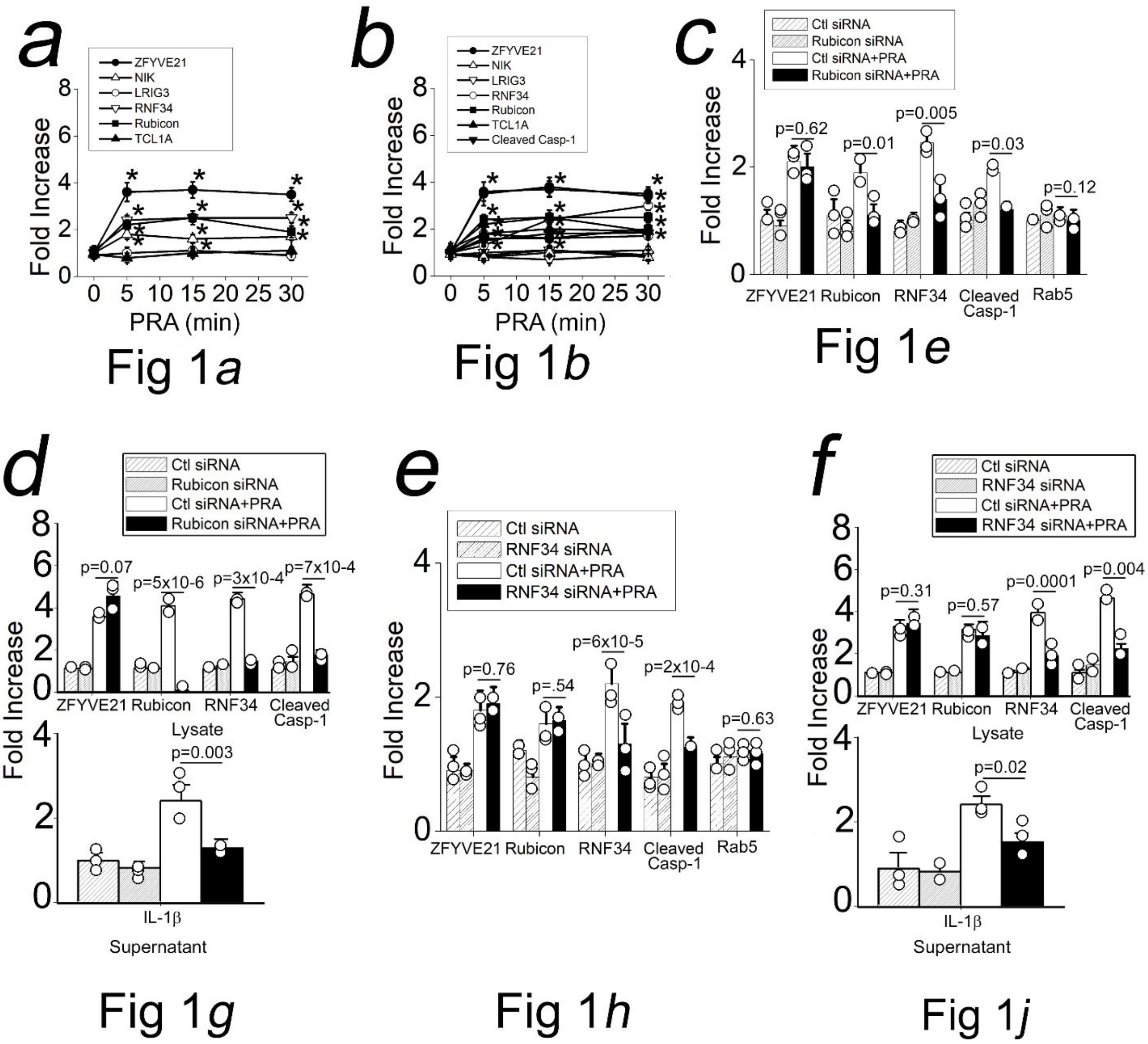

Supplementary Figure 3. Densitometries for Main Fig 1. Densitometries were performed for Western blots and co-IPs in main Fig 1 as indicated. For Supplementary Fig 2*a-c*, # and * indicate $p<0.05$ for Rubicon and RNF34, respectively, compared to timepoint zero using one-way ANOVA followed by Tukey's pairwise comparison. For Supplementary Fig 2*d-e,* one-way ANOVA followed by Tukey's pairwise comparison was used as indicated with non-significant (N.S.) comparisons signifying $p>0.05$. Experiments repeated 2 times (*a-d*) or 4 times (*e,f*). Data are presented as mean values +/- SD.

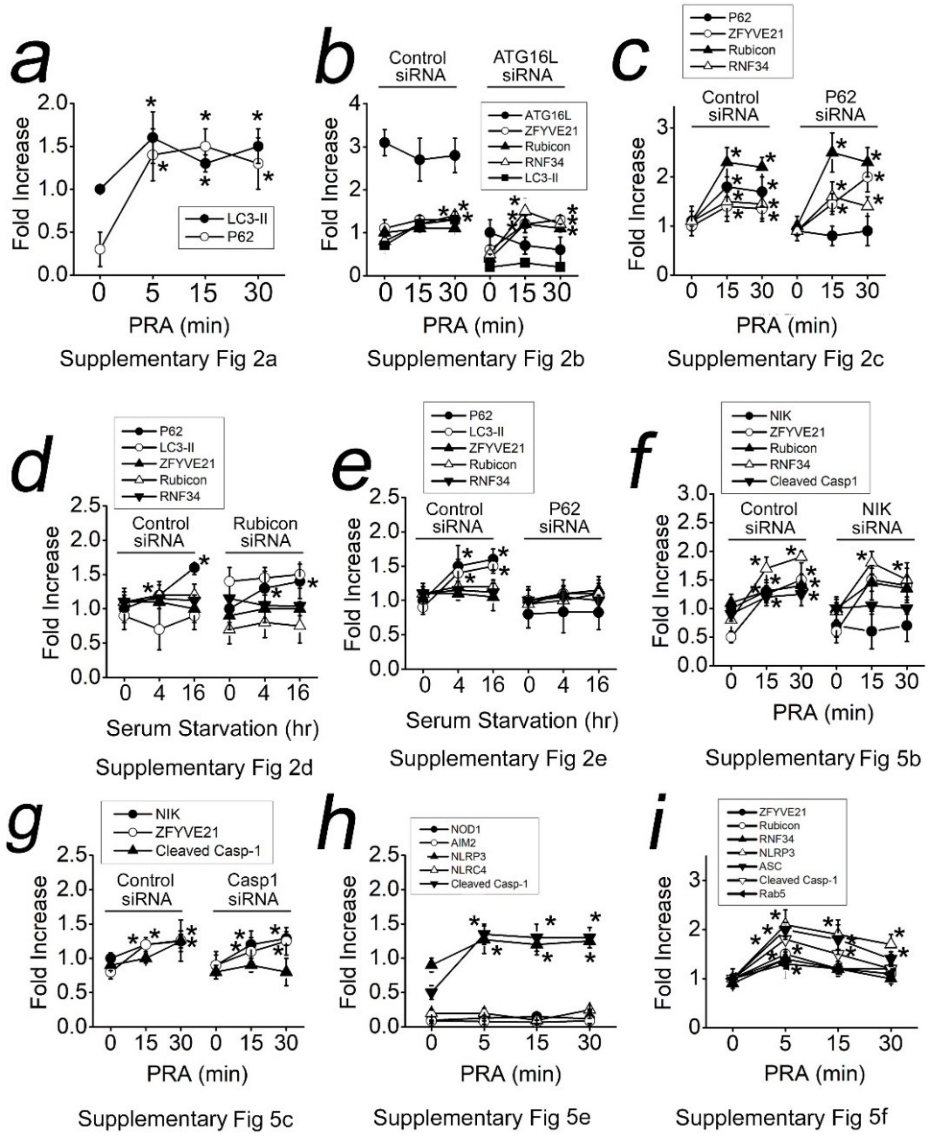

Supplementary Figure 4. Densitometries for Main Fig 2. Densitometries were performed for Western blots and co-IPs in Supplementary Fig 2 and Supplementary Fig 5 as indicated. For Supplementary Fig 4*a*, * indicates p<0.05 for LC3-III and P62 compared to timepoint zero using one-way ANOVA followed by Tukey's pairwise comparison. For Supplementary Fig 4*b-d*, * indicates p<0.05 for ZFYVE21, Rubicon, and RNF34 compared to timepoint zero using one-way ANOVA followed by Tukey's pairwise comparison was used as indicated. For Supplementary Fig 4*e*, * indicates p<0.05 for P62 and LC3-II compared to timepoint zero using one-way ANOVA followed by Tukey's pairwise comparison was used as indicated. For Supplementary Fig 4*f-g*, * indicates p<0.05 for NIK, ZFYVE21, Rubicon, RNF34, and cleaved caspase-1 compared to timepoint zero using one-way ANOVA followed by Tukey's pairwise comparison was used as indicated. For Supplementary Fig 4*h*, * indicates p<0.05 for NLRP3, cleaved caspase-1 compared to timepoint zero using one-way ANOVA followed by Tukey's pairwise comparison was used as indicated. For Supplementary Fig 4*i*, * indicates p<0.05 for ZFYVE21, Rubicon, RNF34, NLRP3, ASC, and cleaved caspase-1 compared to timepoint zero using one-way ANOVA followed by Tukey's pairwise comparison was used as indicated. Experiments were repeated 2 times (*a-i*). Data are presented as mean values +/- SD.

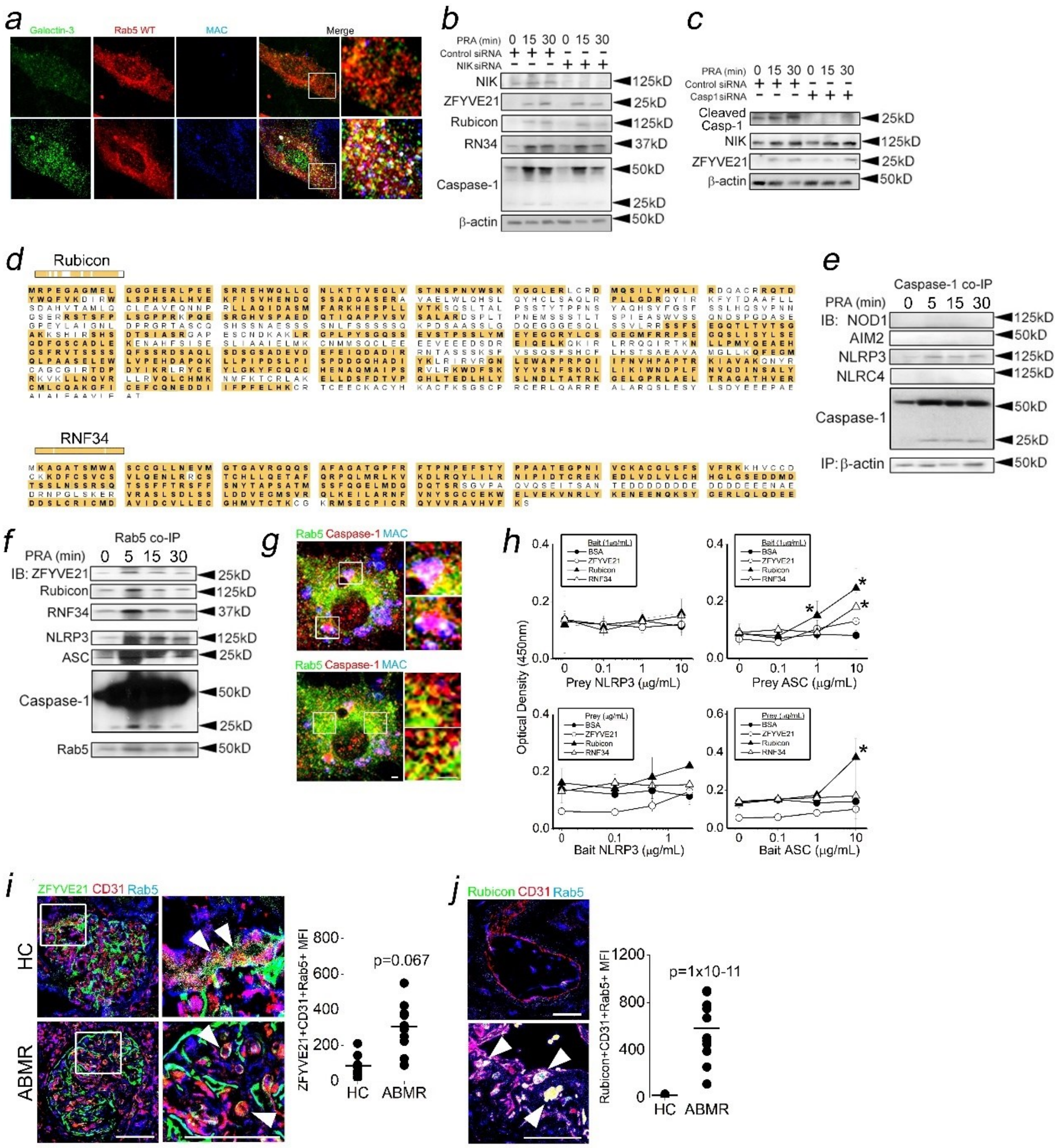

Supplementary Figure 5. HUVECs were stably transduced with Rab5 WT-RFP and treated with PRA for 30 min prior to confocal I.F. analysis (*a*). HUVECs were transfected with siRNA against NIK (*b*) or caspase-1 (*c*) prior to treatment with PRA for the indicated times. Rubicon and RNF34 complexes appearing in the SDS-PAGE gel at ~150 kD in Main Figure 3*d* were analyzed via LC-MS/MS. Regions of peptide coverage by LC-MS/MS are highlighted in yellow (*d*). HUVECs were treated with PRA prior to pulldowns for caspase-1 (*e*) and Rab5 (*f*). HUVECs were transfected with Rab5-GFP and Caspase-1 RFP, treated with PRA, and analyzed by confocal I.F. (*g*). ELISAs were performed to test binding between inflammasome proteins and ZRR complexes using soluble NLRP3 and ASC as prey (top row) and plate-bound NLRP3 and ASC as bait (bottom row, *h*). Human healthy control (HC) and antibody-mediated rejection (ABMR) renal biopsies were analyzed by I.F. (*i*). * represents p<0.05. Two-way ANOVA with Tukey's post-hoc comparison was used for statistical analysis (*h*). Experiments were repeated 2 times (*a-j*) with different HUVEC donors. Experiments repeated 2 times (*a-j*).Data are presented as mean values +/- SD.

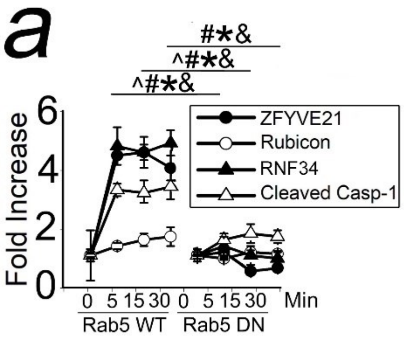

Fig 2*a*

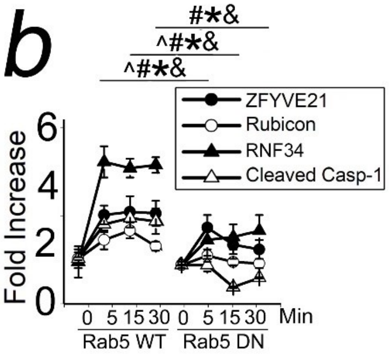

Fig 2*b*

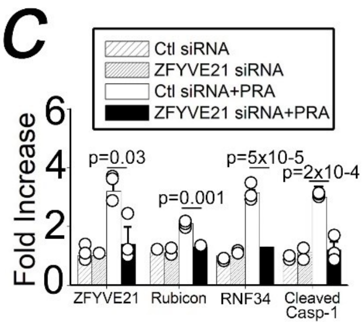

Fig 2*c*

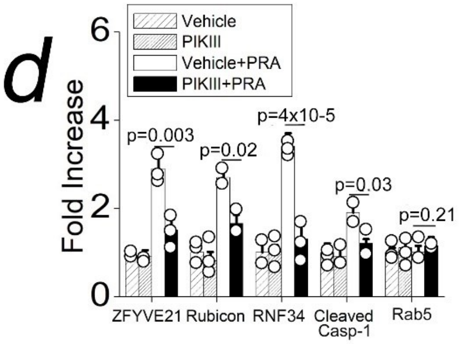

Fig 2*d*

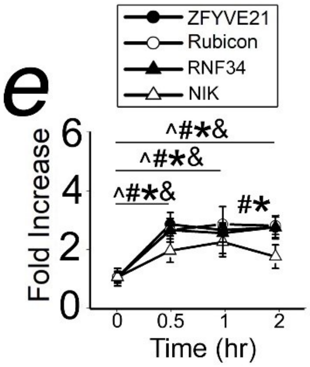

Fig 2*h*

Supplementary Figure 6. Densitometries for Main Fig 2. Densitometries were performed for Western blots and co-IPs in main Fig 2 as indicated. For Supplementary Fig 3*a-b*, ^, #, *, and & indicate $p<0.05$ for ZFYVE21, Rubicon, RNF34, and cleaved caspase-1 respectively, compared to analogous timepoints in Rab5 WT using two-way ANOVA followed by Tukey's pairwise comparison. For Supplementary Fig3*c-d,* one-way ANOVA followed by Tukey's pairwise comparison was used for analysis. For Supplementary Fig 3e, ^, #, *, and & indicate $p<0.05$ for ZFYVE21, Rubicon, RNF34, and NIK compared to timepoint zero. Experiments were repeated 3 times (*a-k*) with different HUVEC donors. Data are presented as mean values +/- SD.

.

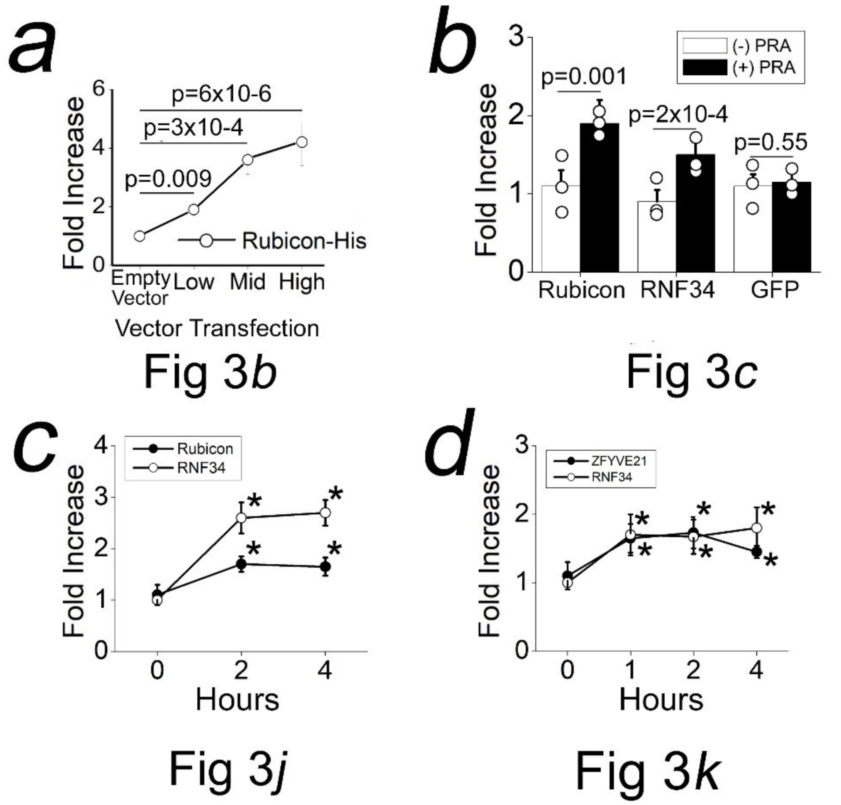

Supplementary Figure 7. Densitometries for Main Fig 3. Densitometries were performed for Western blots and co-IPs in main Fig 3 as indicated. For Supplementary Fig 4*a*, one-way ANOVA followed by Tukey's pairwise comparison was used. For Supplementary Fig 4*b*, Student's *t*-test was used. For Supplementary Fig 4*c*-*d*, * indicates $p<0.05$ compared to timepoint zero using one-way ANOVA followed by Tukey's pairwise comparison. Experiments repeated 2 times (*a*) or 3 times (*b*-*d*) with different HUVEC donors. Data are presented as mean values +/- SD.

.

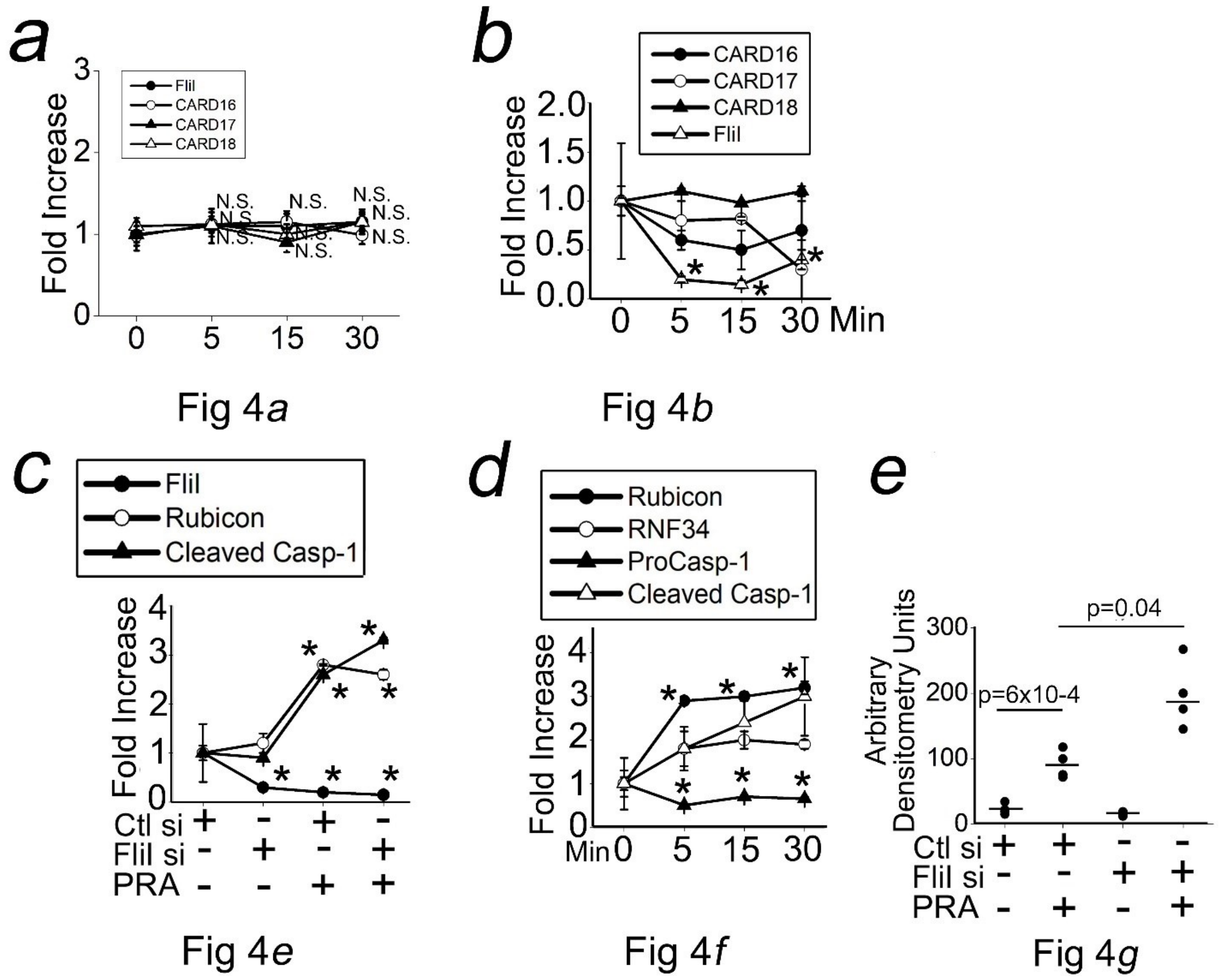

Supplementary Figure 8. Densitometries for Main Fig 4. Densitometries were performed for Western blots and co-IPs in main Fig 4 as indicated. For Supplementary Fig 4*a-d*, * and N.S. indicate $p<0.05$ and $p>0.05$, respectively, compared to timepoint zero using one-way ANOVA followed by Tukey's pairwise comparison. For Supplementary Fig 4*e*, * indicates $p<0.05$ using two-way ANOVA followed by Tukey's pairwise comparison. Experiments repeated 3 times (*a-e*) with different HUVEC donors. Data are presented as mean values +/- SD.

.

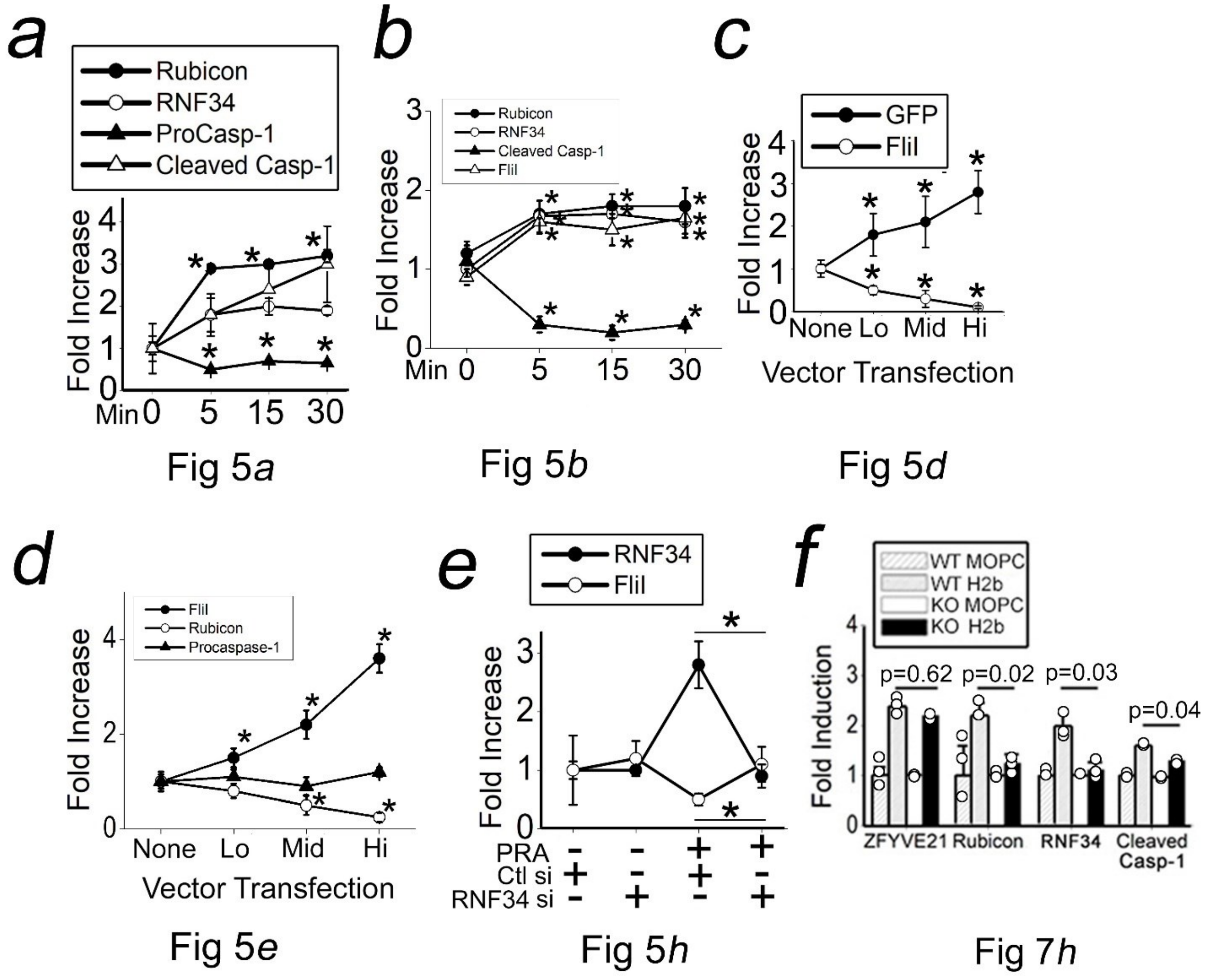

Supplementary Figure 9. Densitometries for Main Fig 5 and Main Fig 7. Densitometries were performed for Western blots and co-IPs in main Fig 5 as indicated. For Supplementary Fig 9*a*-*b*, * indicates $p<0.05$ compared to timepoint zero using one-way ANOVA followed by Tukey's pairwise comparison. For Supplementary Fig 9*c*-*d*, * indicates $p<0.05$ compared to empty vector controls using one-way ANOVA followed by Tukey's pairwise comparison. For Supplementary Fig 9*e*, * indicates $p<0.05$ using one-way ANOVA followed by Tukey's pairwise comparison. For Supplementary Fig 9*f*, * indicates $p<0.05$ compared to the WT MOPC group using one-way ANOVA followed by Tukey's pairwise comparison. Experiments were repeated 3 times with different HUVEC donors. Data are presented as mean values +/- SD.

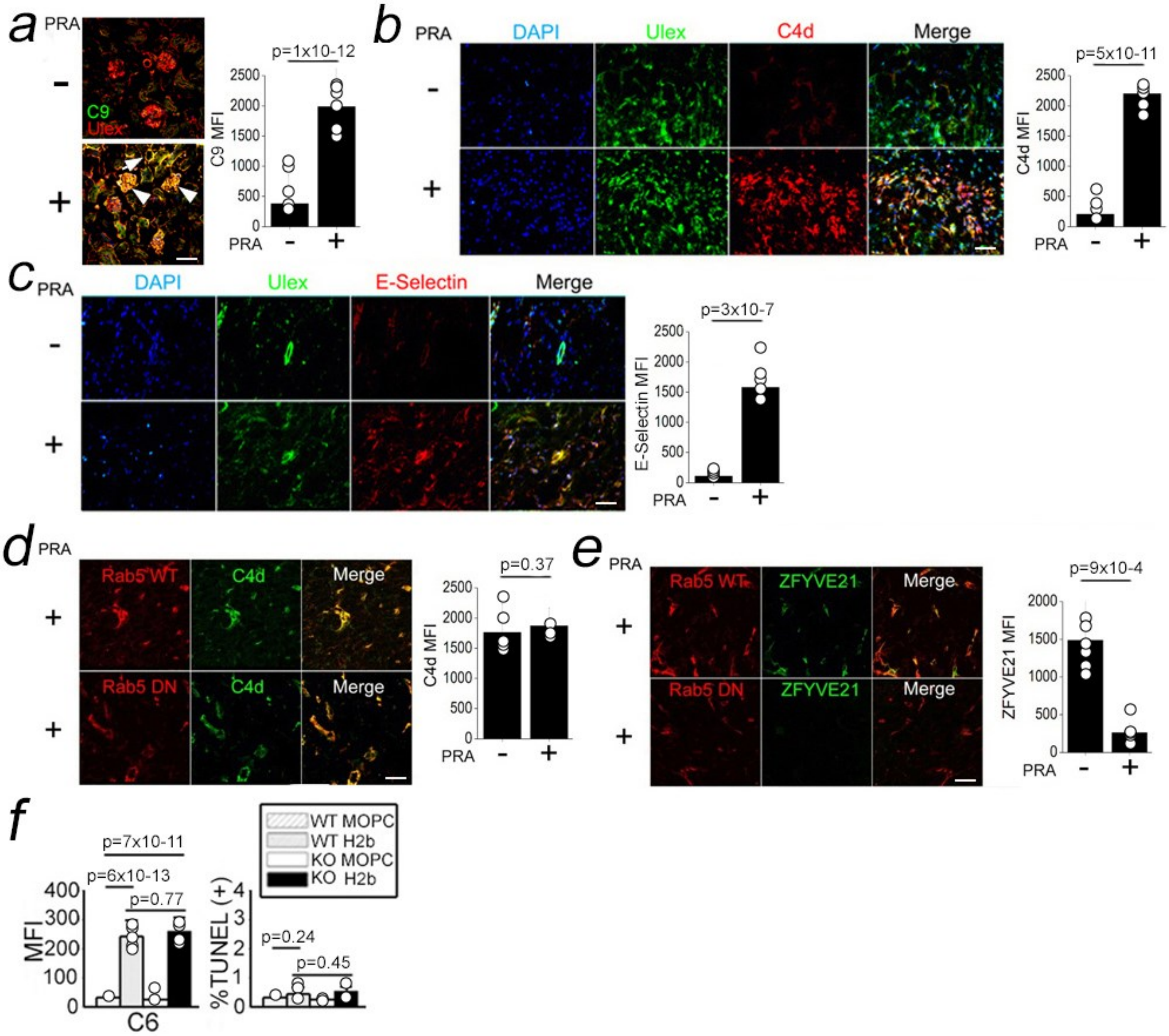

Supplementary Figure 10. ZRR-Mediated Vascular Inflammation *In Vivo.* Confocal I.F. staining of perfused microvessels embedded within collagen gel matrices *in vivo* and treated with or without PRA for 24 hr prior to harvest and I.F. analysis (*a-e*). I.F. analysis of C6 and TUNEL staining of skin from WT or Rubicon-/- hosts treated with MOPC (750μg) or anti-H2^b Ab (750μg) for 24 hr (*f*). n=3 per group (*a-e*), n=6 per group (*f*). Data are presented as mean values +/- SD. * represents p<0.05. Student's *t*-test (*a-e*) and two-way ANOVA with Tukey's post-hoc comparison (*f*) was used for statistical comparisons

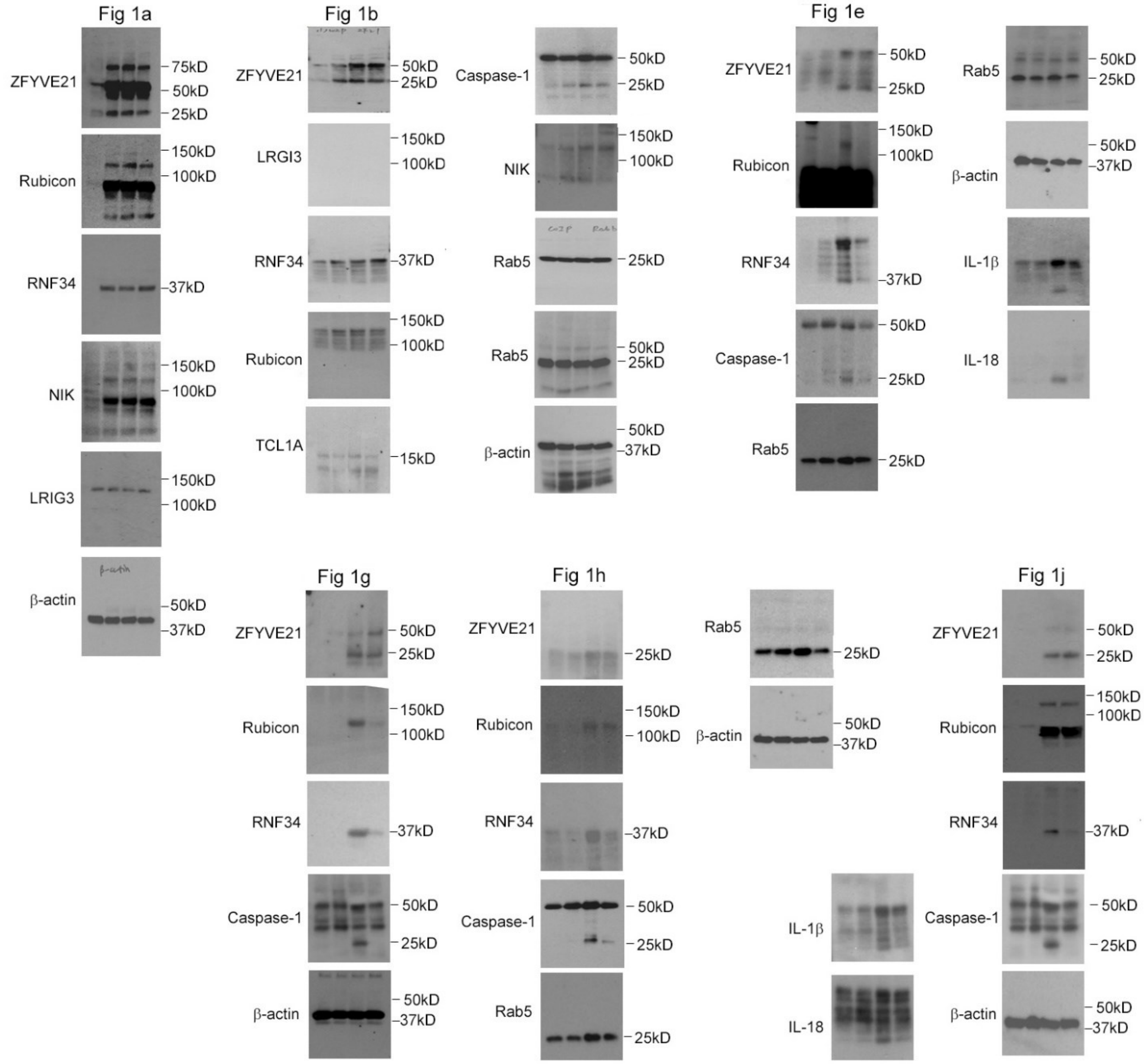

Supplementary Figure 11. Western Blot Films for Main Fig 1. Original uncropped films corresponding to Western blots in main Fig 1 in the manuscript.

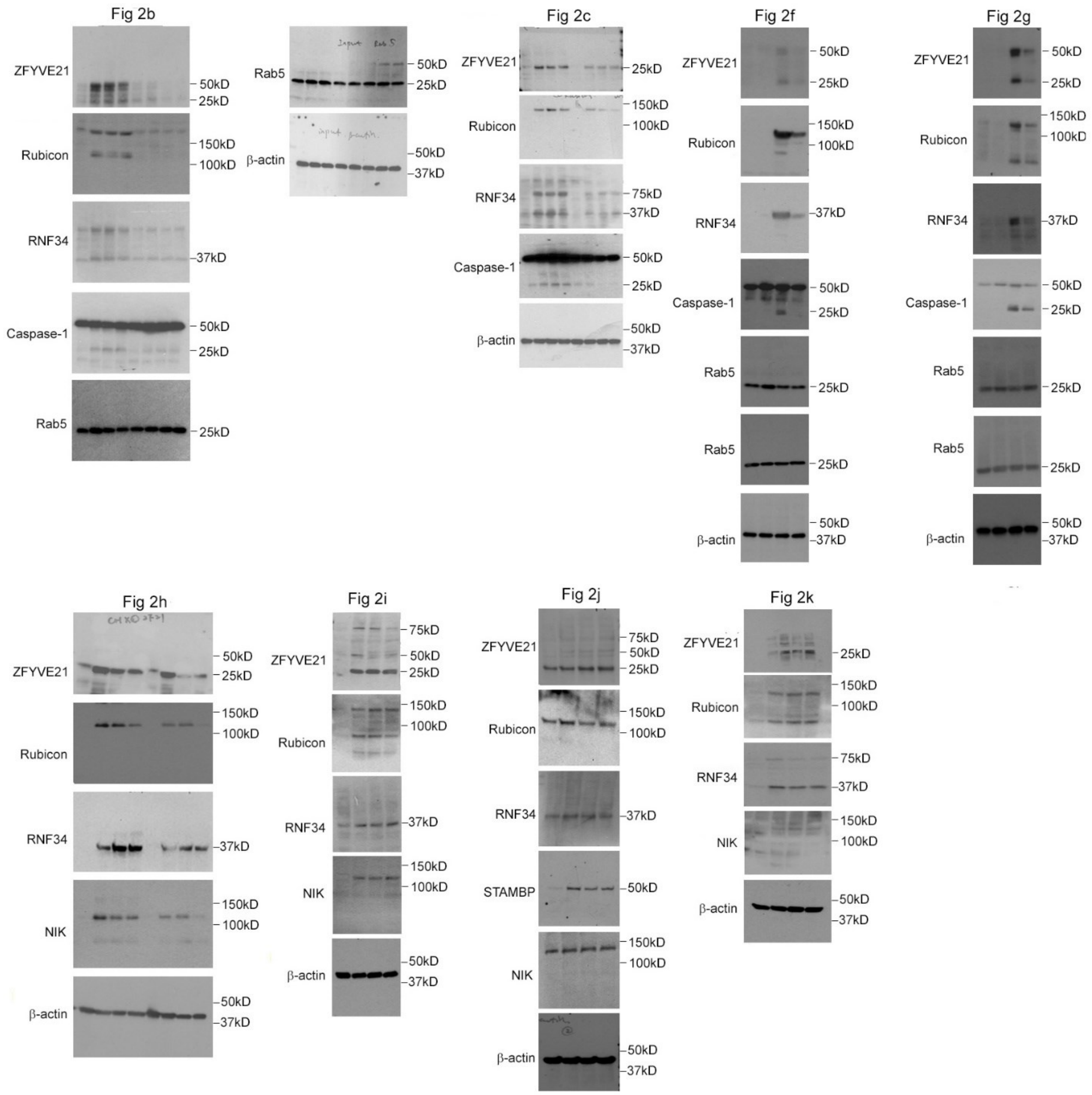

Supplementary Figure 12. Western Blot Films for Main Fig 2. Original uncropped films corresponding to Western blots in Fig 2 of the manuscript.

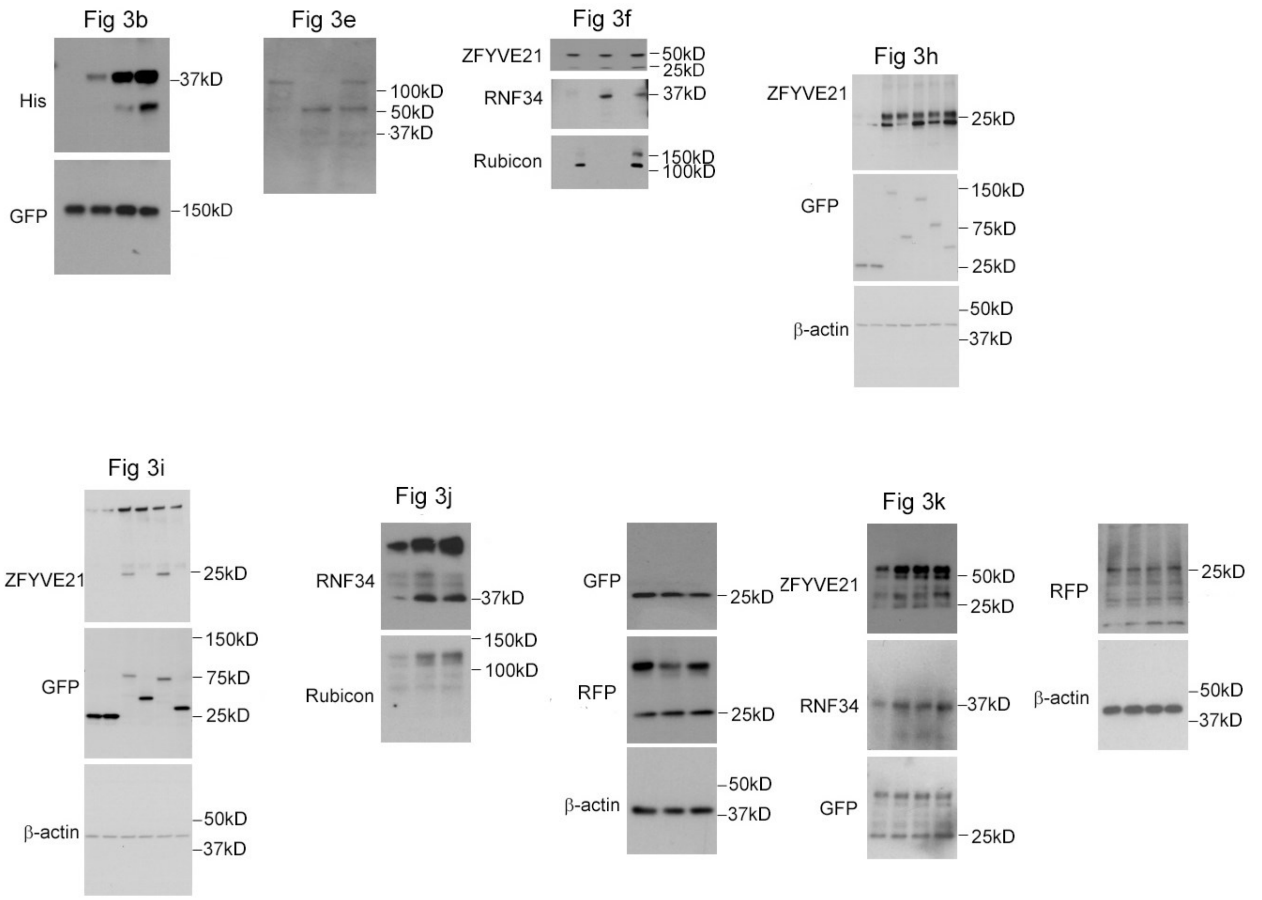

Supplementary Figure 13. Western Blot Films for Main Fig 3. Original uncropped films corresponding to Western blots in Fig 3 of the manuscript.

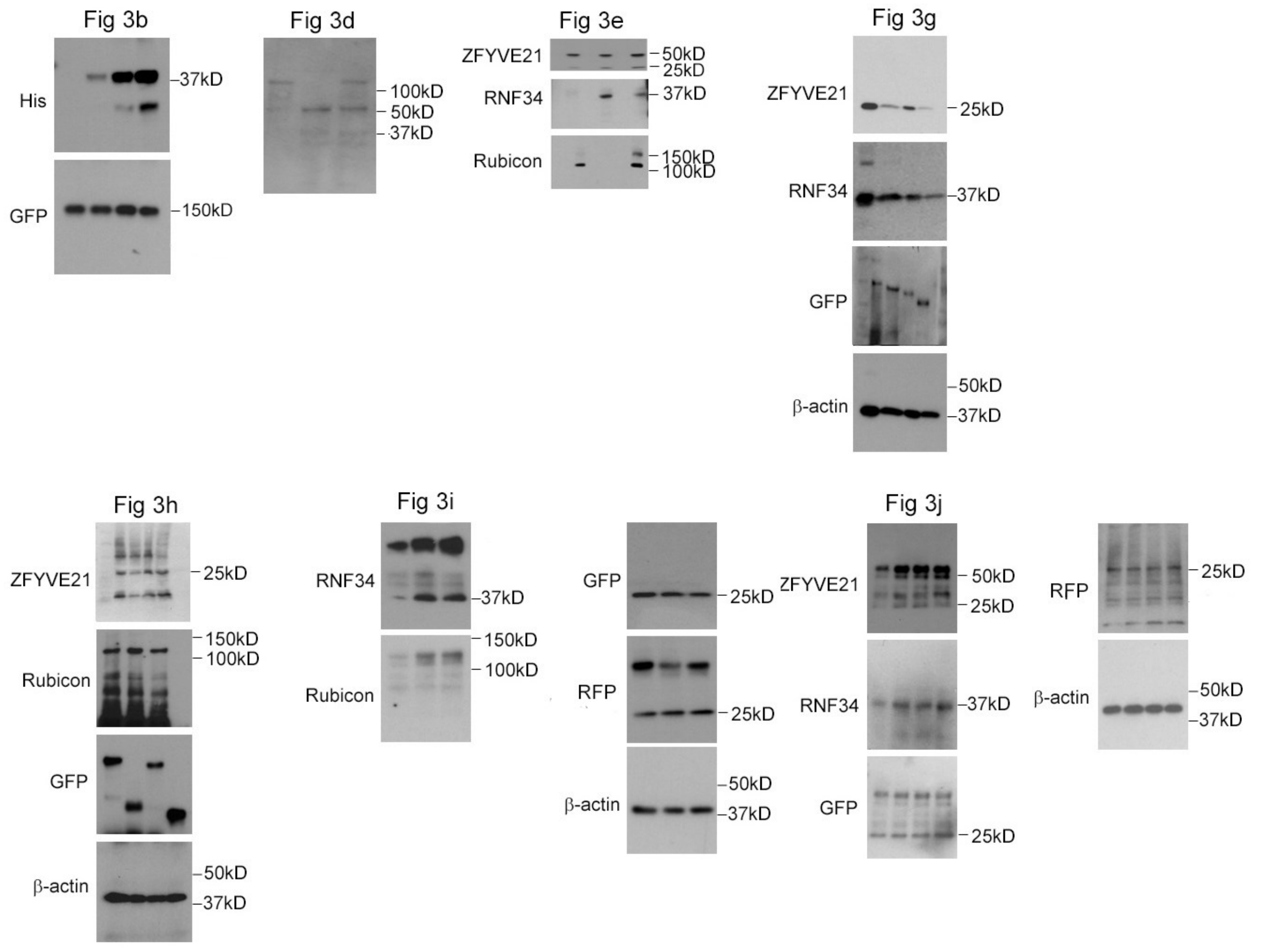

Supplementary Figure 14. Western Blot Films for Main Fig 3. Original uncropped films corresponding to Western blots in Fig 4 of the manuscript.

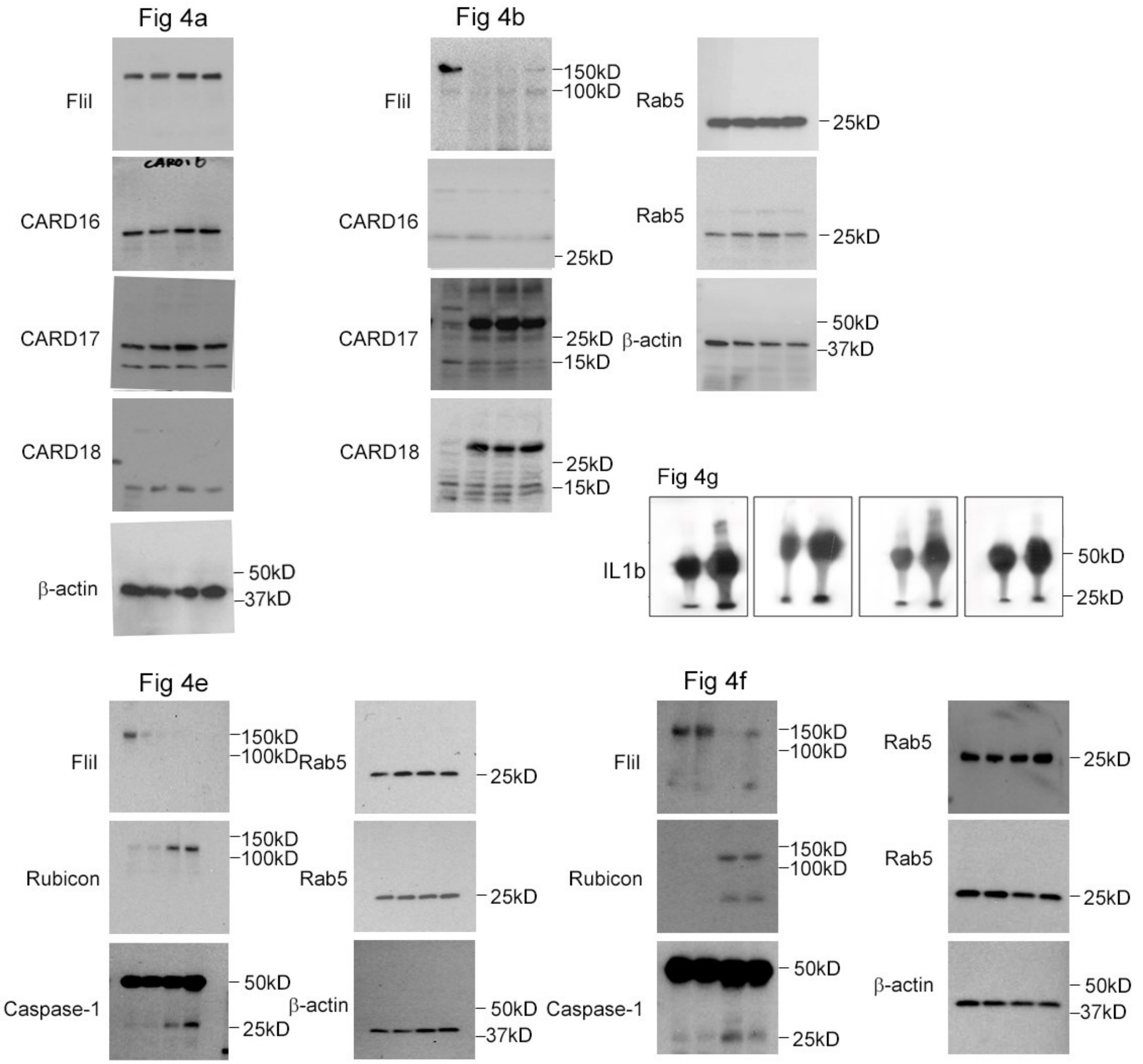

Supplementary Figure 15. Western Blot Films for Main Fig 4. Original uncropped films corresponding to Western blots in Fig 4 of the manuscript.

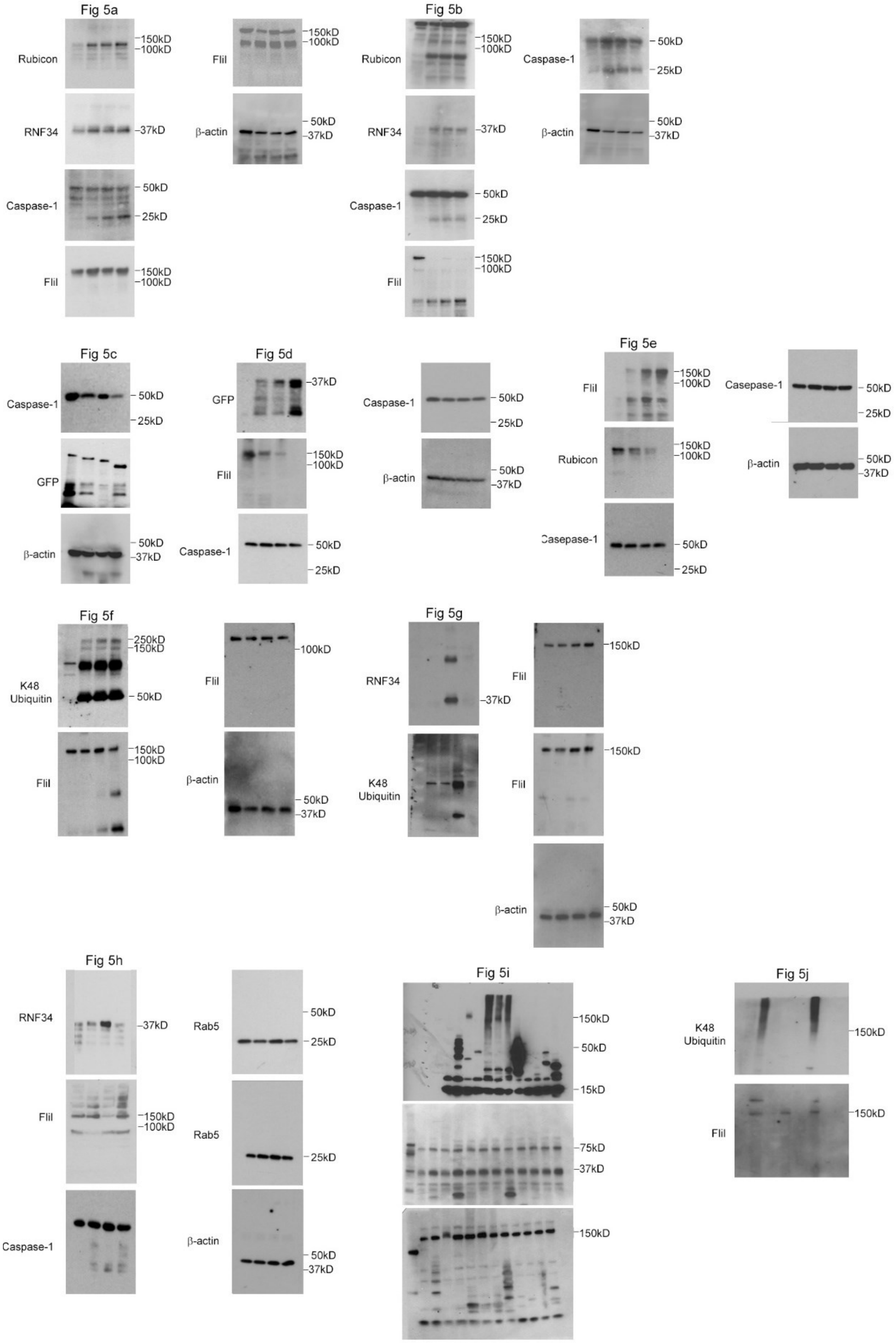

Supplementary Figure 16. Western Blot Films for Main Fig 5. Original uncropped films corresponding to Western blots in Fig 5 of the manuscript.

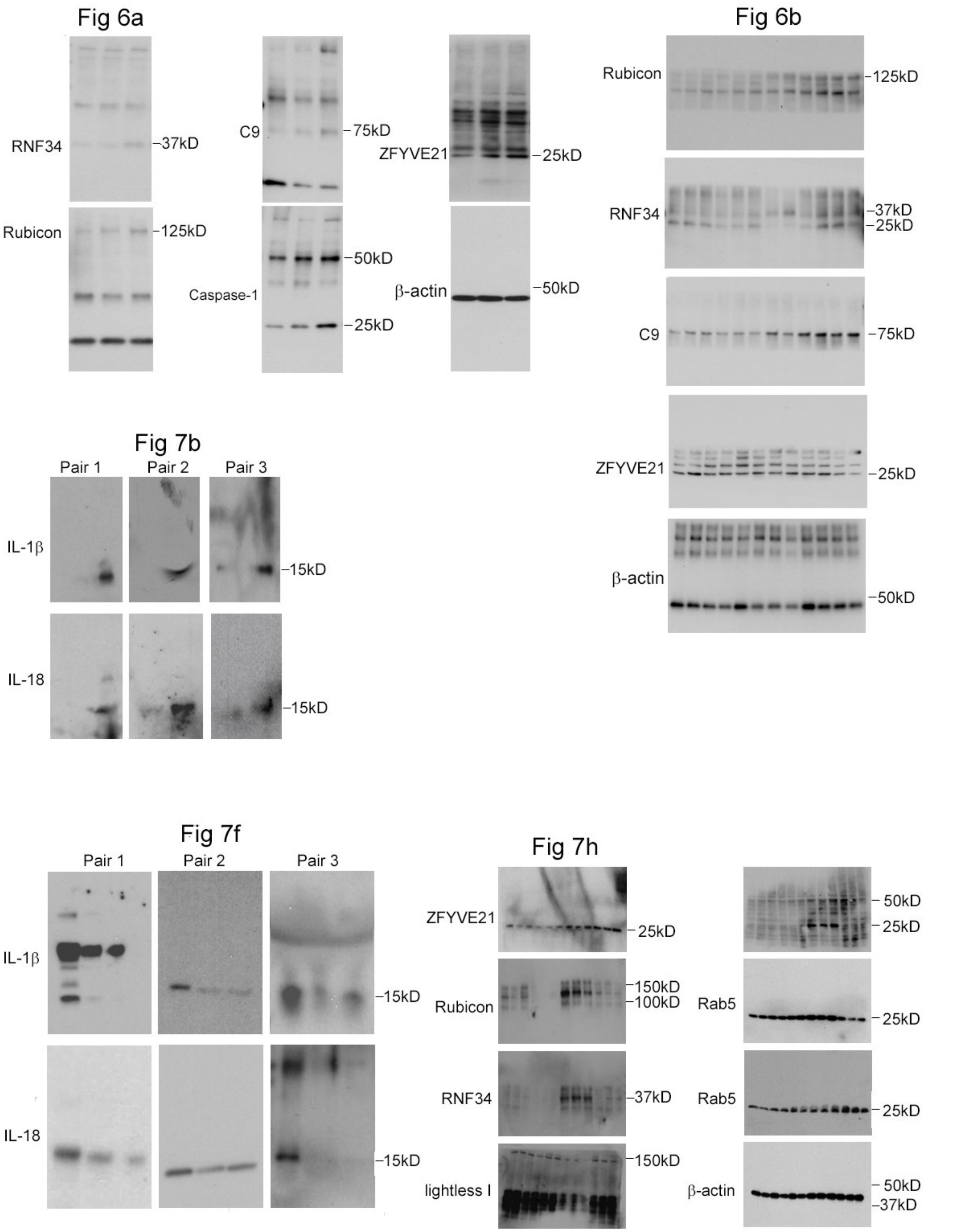

Supplementary Figure S17. Western Blot Films for Main Fig 6 and Main Fig 7. Original uncropped films corresponding to Western blots in Fig 6 and 7 of the manuscript.

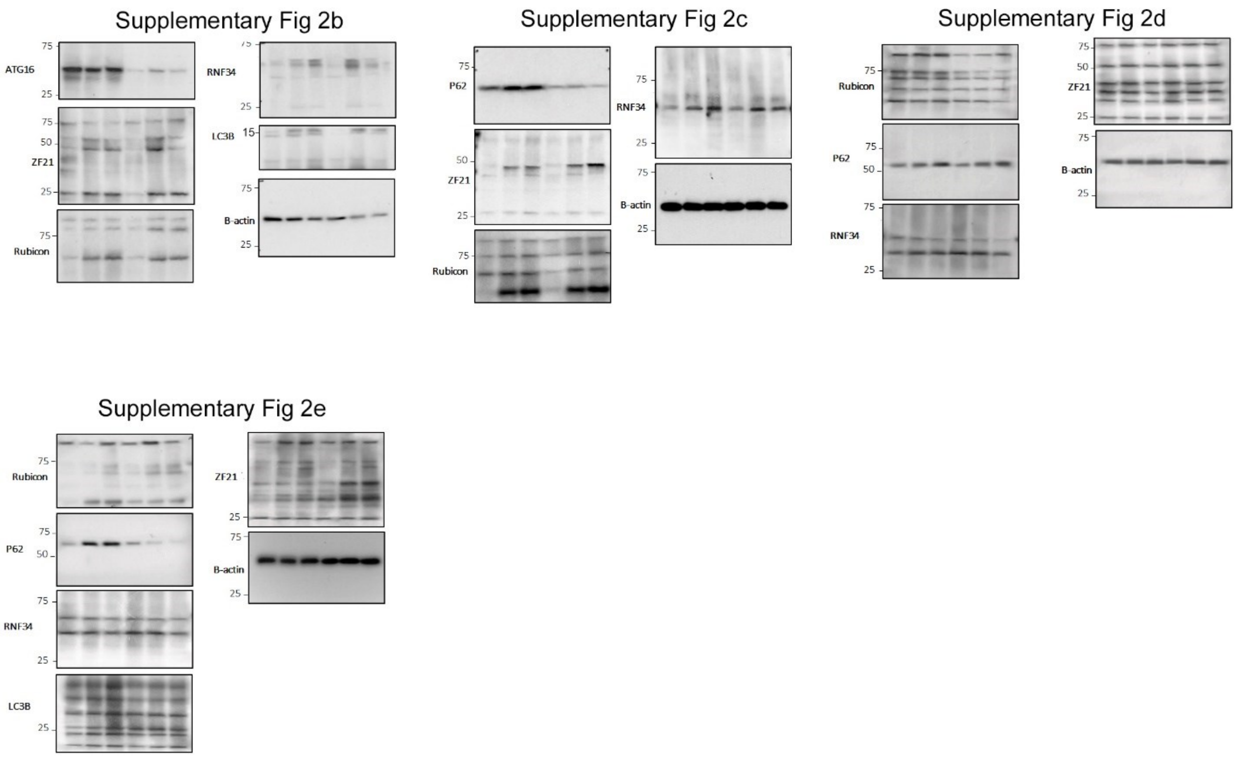

Supplementary Figure S18. Western Blot Films for Supplementary Figure 2. Original uncropped films corresponding to Western blots in Supplementary Fig 5 of the manuscript.

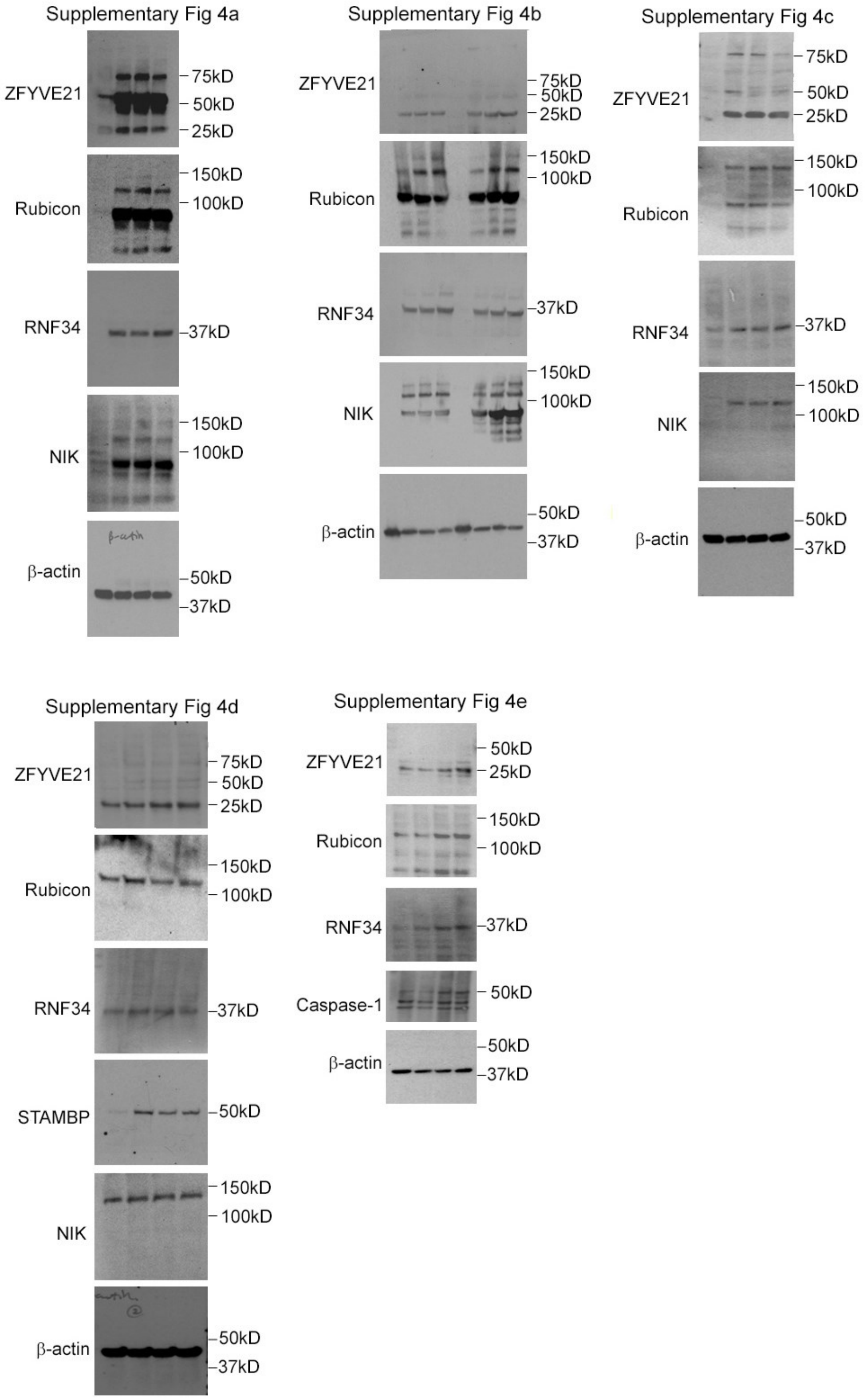

Supplementary Figure S19. Western Blot Films for Supplementary Figure 4. Original uncropped films corresponding to Western blots in Supplementary Fig 4 of the manuscript.

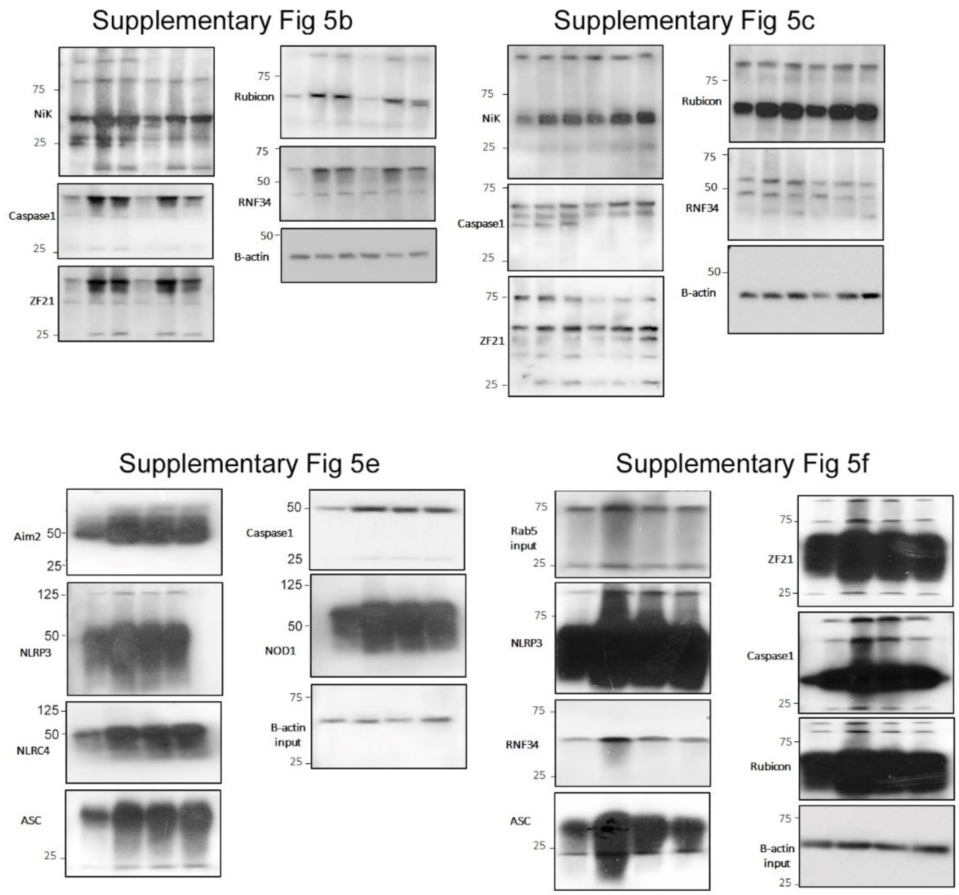

Supplementary Figure S20. Western Blot Films for Supplementary Figure 4. Original uncropped films corresponding to Western blots in Supplementary Fig 5 of the manuscript.
