# Supplementary material for: A ZFYVE21-Rubicon-RNF34 signaling complex promotes endosome-associated inflammasome activity in endothelial cells
Source: Nat Commun. 2023 May 24;14:3002. doi: 10.1038/s41467-023-38684-2 (PMC10209169; doi:10.1038/s41467-023-38684-2)
Supplement: Supplementary file 3 — Reporting Summary [file 41467_2023_38684_MOESM3_ESM.pdf]

nature portfolio

Corresponding author(s): Dan Jane-wit

Last updated by author(s): 1-10-2023

Reporting Summary

Nature Portfolio wishes to improve the reproducibility of the work that we publish. This form provides structure for consistency and transparency in reporting. For further information on Nature Portfolio policies, see our Editorial Policies and the Editorial Policy Checklist.

Statistics

For all statistical analyses, confirm that the following items are present in the figure legend, table legend, main text, or Methods section.

n/a	Confirmed	
☐	☒	The exact sample size (*n*) for each experimental group/condition, given as a discrete number and unit of measurement
☐	☒	A statement on whether measurements were taken from distinct samples or whether the same sample was measured repeatedly
☐	☒	The statistical test(s) used AND whether they are one- or two-sided<br>*Only common tests should be described solely by name; describe more complex techniques in the Methods section.*
☐	☒	A description of all covariates tested
☐	☒	A description of any assumptions or corrections, such as tests of normality and adjustment for multiple comparisons
☐	☒	A full description of the statistical parameters including central tendency (e.g. means) or other basic estimates (e.g. regression coefficient) AND variation (e.g. standard deviation) or associated estimates of uncertainty (e.g. confidence intervals)
☐	☒	For null hypothesis testing, the test statistic (e.g. *F*, *t*, *r*) with confidence intervals, effect sizes, degrees of freedom and *P* value noted<br>*Give P values as exact values whenever suitable.*
☐	☒	For Bayesian analysis, information on the choice of priors and Markov chain Monte Carlo settings
☐	☒	For hierarchical and complex designs, identification of the appropriate level for tests and full reporting of outcomes
☐	☒	Estimates of effect sizes (e.g. Cohen's *d*, Pearson's *r*), indicating how they were calculated

Our web collection on statistics for biologists contains articles on many of the points above.

Software and code

Policy information about availability of computer code

Data collection	N/A
Data analysis	N/A

For manuscripts utilizing custom algorithms or software that are central to the research but not yet described in published literature, software must be made available to editors and reviewers. We strongly encourage code deposition in a community repository (e.g. GitHub). See the Nature Portfolio guidelines for submitting code & software for further information.

Data

Policy information about availability of data

All manuscripts must include a data availability statement. This statement should provide the following information, where applicable:

- Accession codes, unique identifiers, or web links for publicly available datasets
- A description of any restrictions on data availability
- For clinical datasets or third party data, please ensure that the statement adheres to our policy

The datasets generated during and/or analysed during the current study are available from the corresponding author on reasonable request.

Human research participants

Policy information about studies involving human research participants and Sex and Gender in Research.

Reporting on sex and gender	N/A
Population characteristics	N/A
Recruitment	N/A
Ethics oversight	N/A

Note that full information on the approval of the study protocol must also be provided in the manuscript.

Field-specific reporting

Please select the one below that is the best fit for your research. If you are not sure, read the appropriate sections before making your selection.

☒ Life sciences ☐ Behavioural & social sciences ☐ Ecological, evolutionary & environmental sciences

For a reference copy of the document with all sections, see nature.com/documents/nr-reporting-summary-flat.pdf

Life sciences study design

All studies must disclose on these points even when the disclosure is negative.

Sample size	Based on pilot studies using epidermal hyperplasia as a pre-specified readout, preliminary power estimates revealing a change of ~60% indicated that n=6 at a minimum per group was required to avoid type II error at $p<0.05$.
Data exclusions	No data were excluded from the study.
Replication	Attempts for replication using commercial polyclonal antibodies in Fig 1 involving I.F. staining Rubicon and RNF34 were unsuccessful possibly due to batch variation in the commercial suppliers following COVID-19-associated lockdowns. This required use of overexpression constructs to verify colocalization.
Randomization	Sample treatments and murine hosts were selected randomly.
Blinding	I.F. analyses in Fig 8 were performed by three investigators who were blinded to the allocation of specific host treatments.

Reporting for specific materials, systems and methods

We require information from authors about some types of materials, experimental systems and methods used in many studies. Here, indicate whether each material, system or method listed is relevant to your study. If you are not sure if a list item applies to your research, read the appropriate section before selecting a response.

Materials & experimental systems

n/a	Involved in the study	
☐	☒	Antibodies
☐	☒	Eukaryotic cell lines
☒	☐	Palaeontology and archaeology
☐	☒	Animals and other organisms
☐	☒	Clinical data
☒	☐	Dual use research of concern

Methods

n/a	Involved in the study	
☒	☐	ChIP-seq
☐	☒	Flow cytometry
☒	☐	MRI-based neuroimaging

Antibodies

Antibodies used	All antibody information including supplier, catalog number, and dilution have been provided in the Materials & Methods section of the manuscript.
Validation	All antibodies tested were validated by the supplier and tested using relevant siRNA knockdowns. As noted above, certain commercial polyclonal antibodies for Rubicon and RNF34 failed replication following COVID-19-related lockdowns.

Eukaryotic cell lines

Policy information about cell lines and Sex and Gender in Research

Cell line source(s)	All human umbilical vein endothelial cells (HUVECs) were obtained from female donors.
Authentication	HUVECs were visually inspected for proper morphology, tested for adherence to collagen-coated plates, and stained with Ulex to verify cell identity.
Mycoplasma contamination	All primary and cell line used in the study were tested for Mycoplasma contamination.
Commonly misidentified lines (See ICLAC register)	N/A

Animals and other research organisms

Policy information about studies involving animals; ARRIVE guidelines recommended for reporting animal research, and Sex and Gender in Research

Laboratory animals	Wild-type C567/Bl6 mice and Rubicon-/- mice aged 6-12 weeks old were used in the study.
Wild animals	N/A
Reporting on sex	To elicit chronic rejection, we used male skin grafts expressing H-Y as a target antigen for female splenocytes. As such, we are unable to disaggregate male vs female gender bias in our model system.
Field-collected samples	N/A
Ethics oversight	All relevant animal protocols were approved by the Yale IACUC.

Note that full information on the approval of the study protocol must also be provided in the manuscript.

Clinical data

Policy information about clinical studies

All manuscripts should comply with the ICMJE guidelines for publication of clinical research and a completed CONSORT checklist must be included with all submissions.

Clinical trial registration	N/A
Study protocol	N/A
Data collection	N/A
Outcomes	N/A

Flow Cytometry

Plots

Confirm that:

☒ The axis labels state the marker and fluorochrome used (e.g. CD4-FITC).

☒ The axis scales are clearly visible. Include numbers along axes only for bottom left plot of group (a 'group' is an analysis of identical markers).

☒ All plots are contour plots with outliers or pseudocolor plots.

☒ A numerical value for number of cells or percentage (with statistics) is provided.

Methodology

Sample preparation	Methodologies for FACS-assisted sorting of Rab5+ASC+ForwardScatterHi events are described in the Materials & Methods section of the manuscript.
Instrument	FACS Aria was used for cell sorting and LSR II was used for flow cytometry analyses.
Software	FlowJo software was used to analyze flow cytometry data.
Cell population abundance	Data regarding cell population abundance and gating strategies are shown in Figure S1.

Gating strategy

The gating strategy for FACS sorting of inflammasomes is shown in Figure S1.

☒ Tick this box to confirm that a figure exemplifying the gating strategy is provided in the Supplementary Information.
